# ﻿First record of the genus *Touranella* Attems, 1937 (Diplopoda, Polydesmida, Paradoxosomatidae) from Laos, with a description of a new species

**DOI:** 10.3897/zookeys.1145.98704

**Published:** 2023-02-03

**Authors:** Anh D. Nguyen, Petra Sierwald, Stephanie Ware

**Affiliations:** 1 Institute of Ecology and Biological Resources, Vietnam Academy of Science and Technology, 18, Hoangquocviet Road, Caugiay District, Hanoi, Vietnam Institute of Ecology and Biological Resources, Vietnam Academy of Science and Technology Hanoi Vietnam; 2 Graduate University of Science and Technology, Vietnam Academy of Science and Technology, 18, Hoangquocviet Road, Caugiay District, Hanoi, Vietnam Graduate University of Science and Technology, Vietnam Academy of Science and Technology Hanoi Vietnam; 3 Field Museum of Natural History, 1400 S. Lakeshore Drive, 60605, Chicago, IL, USA Field Museum of Natural History Chicago United States of America

**Keywords:** Bioinventory, Champasak, diversity, millipede, new species, taxonomy

## Abstract

The paradoxosomatid genus *Touranella* Attems, 1937 is recorded from Laos for the first time, with a new species, *Touranellachampasak***sp. nov.**, described here. The taxonomy of the genus is discussed, an identification key is provided, and the current distribution of all species is mapped.

## ﻿Introduction

The genus *Touranella* Attems, 1937 was established for a single species, *Touranellagracilis* Attems, 1937. Attems (1937) distinguished this genus from other paradoxosomatids recorded in Vietnam at that time by the following features: gonopod femorite strongly reduced or completely absent, and solenomere (= Rinnenast) arising from the prefemorite. No other records had been reported until Golovatch (1994: 186) described the second species of the genus, *Touranellahimalayaensis* Golovatch, 1994, from Nepal. The type locality of *T.himalayaensis* lies approximately 2,500 km north of Vietnam, indicating a significant biogeographical gap among species in the same genus. Six additional species of *Touranella* were described between 2009–2018, five from Vietnam and one from Nepal. To date, eight *Touranella* species have been described (Sierwald and Spelda 2021), which are listed below:

1. *Touranellacattiensis* Golovatch & Semenyuk, 2010 from Cat Tien National Park, Dong Nai, Vietnam.
2. *Touranellagracilis* Attems, 1937 from Da Nang, Vietnam.
3. *Touranellahimalayaensis* Golovatch, 1994 from Panchthar, Nepal.
4. *Touranellahirsuta* Golovatch, 2009 from Bi Doup–Nui Ba National Park, Lam Dong, Vietnam.
5. *Touranellamoniliformis* Golovatch & Semenyuk, 2018 from Cat Tien National Park, Dong Nai, Vietnam.
6. *Touranellapeculiaris* Golovatch, 2009 from Bi Doup–Nui Ba National Park, Lam Dong, Vietnam.
7. *Touranellapilosa* Golovatch, 2016 from Sankhua Sabha, Nepal.
8. *Touranellatrichosa* Golovatch & Semenyuk, 2018 from Kon Ka Kinh National Park, Gia Lai, Vietnam.

**Figure 1.**
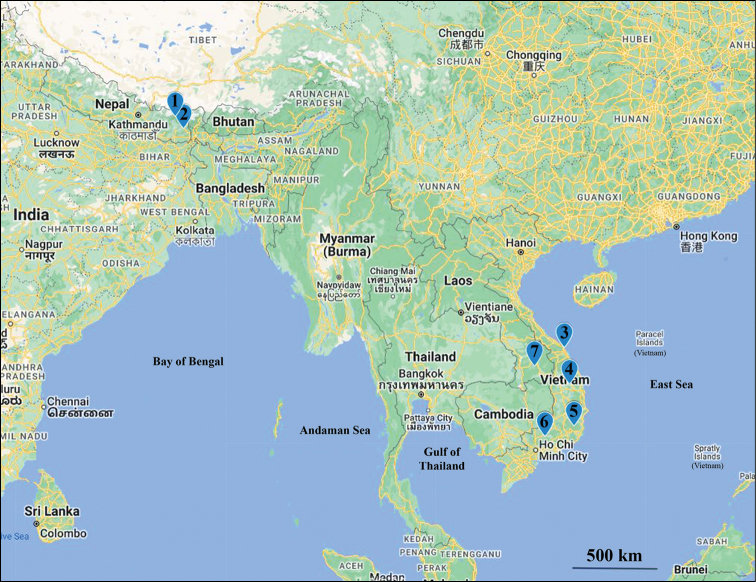
Distribution of the genus *Touranella* Attems, 1937. 1 = *Touranellapilosa* Golovatch, 2016, 2 = *Touranellahimalayaensis* Golovatch, 1994, 3 = *Touranellagracilis* Attems, 1937, 4 = *Touranellatrichosa* Golovatch & Semenyuk, 2018, 5 = *Touranellahirsuta* Golovatch, 2009 and *Touranellapeculiaris* Golovatch, 2009, 6 = *Touranellacattiensis* Golovatch & Semenyuk, 2010 and *Touranellamoniliformis* Golovatch & Semenyuk, 2018, 7 = *Touranellachampasak* sp. nov.

This work reports the first record of *Touranella* in Laos, with a description of a new species. With this discovery, the geographical gap in the distribution of this genus is slightly narrowed (Fig. 1).

## ﻿Material and methods

Examined material was collected by M. Thayer and her colleagues during their field expedition to Laos in 2008 and is currently housed in the Field Museum of Natural History (FMNH).

The specimen was examined under a Leica M205 microscope. Line drawings were made using a camera lucida attached to the Leica M205 microscope. Colour images were taken using the Nikon 5100 imaging system with varying lens sizes under normal and ultraviolet (UV) light. Images were photographed in different layers and stacked using Helicon Focus v. 6.0, then grouped into plates in Photoshop v. 6.0. A gonopod was dissected for morphological observation and mounted on an aluminum stub, coated with gold for SEM imaging. SEM images were taken using a Leo Scanning Electron Microscope (Carl Zeiss SMT, Peabody, MA) at FMNH. A distribution map was created using Google Map.

### ﻿Abbreviations

**FMNH** Field Museum of Natural History;

**INS** Insect Division;

**NP** National Park.

## ﻿Taxonomic part

### ﻿Order Polydesmida Pocock, 1887

**Family Paradoxosomatidae Daday, 1889**

#### ﻿Genus Touranella

Taxon classificationAnimaliaPolydesmidaParadoxosomatidae

Attems, 1937

BB864A87-C736-5B0F-BECE-02CC20864090

Touranella Attems, 1937: 231.
Touranella —Attems 1938: 233; Hoffman 1963: 591 (placed in the newly described tribe Alogolykini); Jeekel 1968: 64, “*incertae sedis*”; Hoffman 1980: 172, “unassigned tribal position”; Golovatch 1994: 187 (placed in the Alogolykini); Golovatch 2009a: 6; Golovatch 2009b: 120; Nguyen and Sierwald 2013: 1179; Golovatch 2016: 139; Golovatch and Semenyuk 2018: 16.

##### Type species.

*Touranellagracilis* Attems, 1937, by original designation.

#### ﻿ Touranella champasak sp. nov.

Taxon classificationAnimaliaPolydesmidaParadoxosomatidae

1F34CB14-33CE-572D-9DB7-026C09E2004D

https://zoobank.org/09F5D34D-0F66-44F5-A53B-CE90DB8B0244

**Figure 2.**
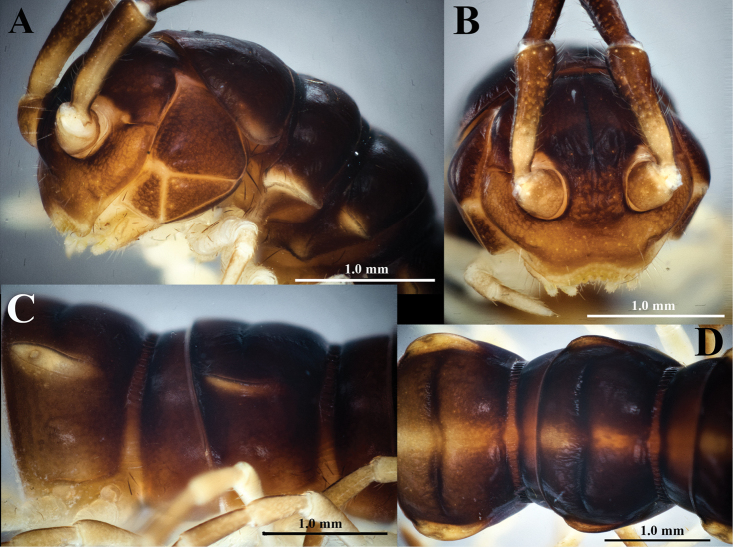
*Touranellachampasak* sp. nov., holotype **A, B** head, lateral and anterior views, respectively **C, D** segments 8 and 9, lateral and dorsal views, respectively.

**Figure 3.**
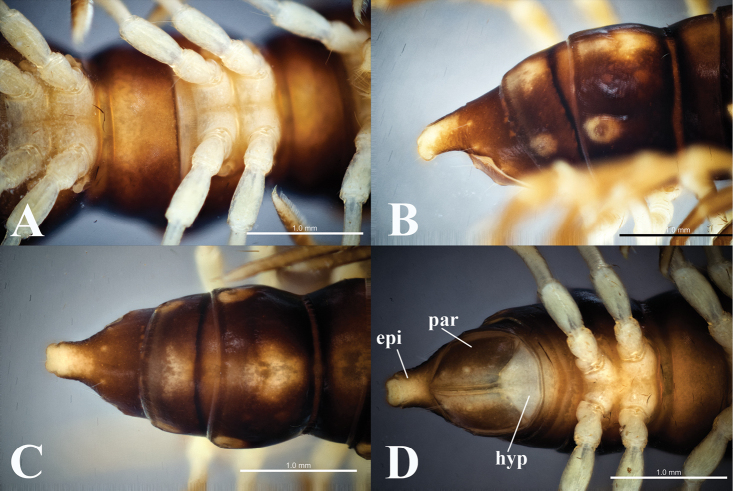
*Touranellachampasak* sp. nov., holotype **A** segments 8 and 9, ventral view **B–D** caudal part of body, lateral, dorsal, and ventral views, respectively. Abbreviations: epi = epiproct; par = paraproct; hyp = hypoproct.

**Figure 4.**
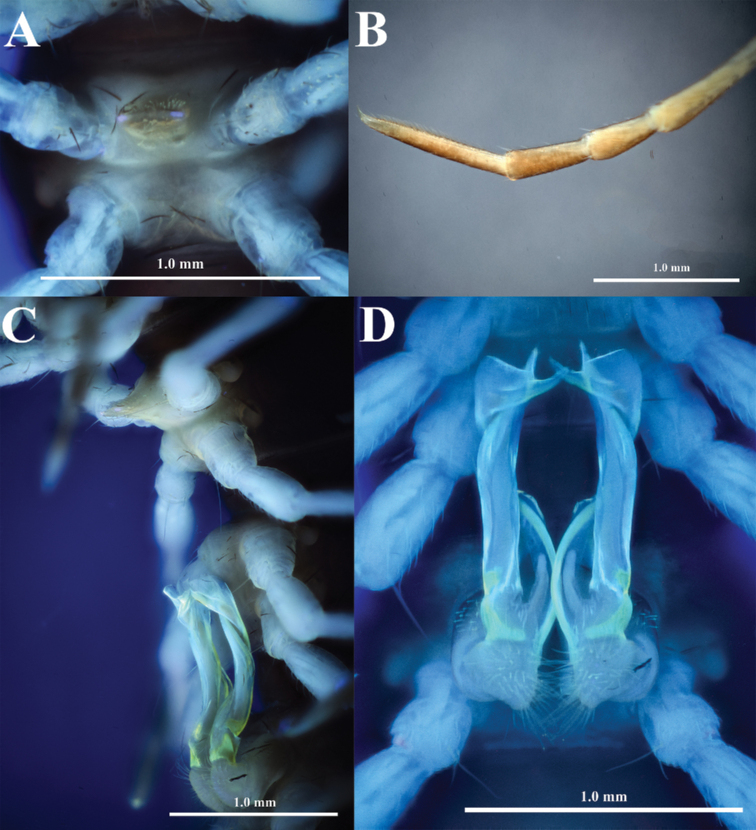
*Touranellachampasak* sp. nov., holotype **A** sternum 5, ventral view, UV light **B** posterior leg on segment 9, anterior view, normal light **C** gonopods and sternum 5, lateral view, UV light **D** gonopods, ventral view, UV light.

**Figure 5.**
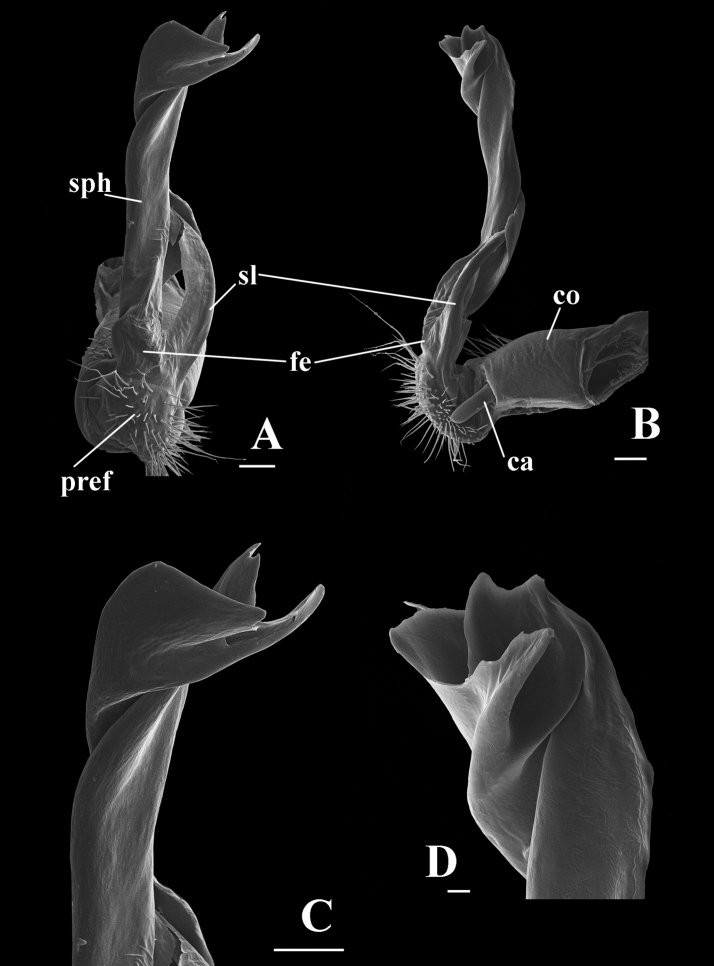
*Touranellachampasak* sp. nov., holotype **A, B** right gonopod, ventral view and mesal view, respectively **C, D** distal part of gonopod, ventral view and mesal view, respectively. Abbreviations: co = gonocoxite; pref = gonoprefemorite; fe = gonofemorite; sph = solenophore; sl = solenomere; ca = cannula. Scale bars: 0.1 mm (**A–C**), 0.02 mm (**D**).

**Figure 6.**
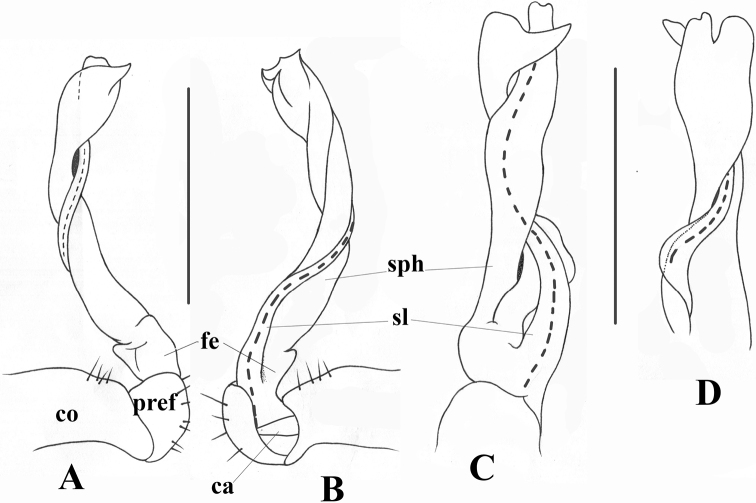
*Touranellachampasak* sp. nov., holotype **A–D** right gonopod, lateral, mesal, dorsal, and ventral views, respectively. Abbreviations: co = gonocoxite; pref = gonoprefemorite; fe = gonofemorite; sph = solenophore; sl = solenomere; ca = cannula. Scale bars: 1 mm.

Figs 2 , 3 , 4 , 5 , 6

##### Material examined.

***Holotype***: Laos • male; Champasak Province, Bolaven Plateau, Ban Thongvay (=Xekatam), vic. old logging road, N of village; 15°14.288'N, 106°31.891'E; 1,095 m elev.; 8–16 June 2008; A. Newton & M. Thayer leg.; selectively logged forest, FMHD#2008-037, flight intercept trap, ANMT site 1231; **FMNHINS 3716303**.

##### Diagnosis.

The new species can be recognized by a submoniliform body; poorly developed paraterga; sparsely setose metaterga; the presence of a highly elevated, setose, trapeziform, sternal process between male coxae 4; a strongly reduced gonofemorite devoid of a femoral process; a somewhat twisted solenophore that distally sheaths a rod-shaped solenomere; and well-developed lamina medialis and lamina lateralis.

The species is most similar to *Touranellamoniliformis* Golovatch & Semenyuk, 2018 from Cat Tien NP (Vietnam) by having a (sub-)moniliform body, poorly developed paraterga, and sparsely setose metaterga. The two species can be distinguished by the gonopod conformation, and the presence of a gonofemoral process in *T.moniliformis* (absent from the new species).

Regarding the absence of a gonofemoral process, the new species is similar to *T.peculiaris* Golovatch, 2009, but can be distinguished by a strongly reduced gonofemorite (vs considerably elongated in *T.peculiaris*).

##### Etymology.

The species epithet, “*champasak*”, is a noun in apposition and refers to the province name where the type was collected.

##### Description.

Holotype length ca 21.6 mm, width of midbody pro- and metazona about 1.5 mm and 1.9 mm, respectively.

Body brown and darkish brown, except several antennomeres; legs and sterna brownish yellow or yellow; posterior margins of prozonae and metazonae, anterior margins of metazonae, and transverse sulcus black; metaterga with a yellow axial band running from collum to telson (Fig. 2D).

Antenna long and slender, approximately reaching to segment 5 when extended back; antennomere 1 very short and robust (Fig. 2B); antennomere 2=3=4=5=6> 7 in length. Tip with four sensory cones. Antennomere 2 strongly constricted at base (Fig. 2A, B).

Collum smooth and shiny, suboval, with two rows of setae: 3+3 anterior and 2+2 posterior. Paraterga small, broadly rounded lobe (Fig. 2A).

Body submoniliform. Prozonae and metazonae smooth, shiny (Figs 2A, C–D, 3). Metatergal transverse sulcus present from segment 5, but completely developed starting on bodyring 6 (Fig. 2D). Metaterga with traces of two setal rows: 2+2 anterior and 2+2 posterior. Pleurosternal carinae present as full crests on segments 2–4, becoming less developed on subsequent segments, completely missing on segments 18–19. Stricture between pro- and metazonae very distinct, fully striolate at bottom on both dorsal and lateral sides (Fig. 3D). Axial line thin, distinct.

Paraterga (Figs 2C, D, 3B, C) yellowish, small as complete crests from lateral side, but more obvious on pore-bearing segments, slightly directed caudally upwards.

Epiproct (**epi**) (Fig. 3B–D) long, broadly truncated, flattened dorsoventrally, lateral tubercles minute; tip with four spinnerets. Hypoproct (**hyp**) (Fig. 3D) subtrapeziform, with two separated, distolateral, setiferous knobs. Paraprocts (**par**) sub-semicircular with two distinct setiferous knobs.

Legs long and slender, about 1.7–1.8 times as long as midbody height. Prefemora not swollen. Femora without modification. Tarsal brushes (Fig. 4B) present on legs until segment 16.

Sterna (Fig. 3A, D) with distinct cross-impression, without modifications except a highly elevated, setose trapeziform process between coxae 4 (Fig. 4A, C). This process carrying a setal brush on anterior side and two pores at base.

Gonopods (Figs 4C, D, 5, 6) simple. Coxite (**co**) subcylindrical, as long as about ½ telopodite, distoventral part sparsely setose. Prefemorite (**pref**) short, densely setose. Femorite (**fe**) strongly reduced, without femoral process. Postfemoral region extremely long, consisting of only solenomere (**sl**) and solenophore (**sph**). Solenomere rod-shaped, arising from prefemorite, distal part sheathed by solenophore, which is suberect, slightly twisted at distal part; lamina lateralis with an apical spine and a well-developed, rounded lobe. Tip of gonopod serrated with three distinct denticles.

##### Remarks.

Even though the distributional gap is slightly narrowed by the occurrence of this genus in Laos, more species most probably have yet to be discovered, at least in and between southern Vietnam and Nepal, including Laos, northern Thailand, and Myanmar (Fig. 1).

### ﻿An identification key to *Touranella* species

Since the recent key provided by Golovatch (2016), three more species have been discovered; therefore, the key is updated.

**Table:** 

1	Metaterga smooth, without setae or with two setal rows	**2**
–	Metaterga with three setal rows or densely setose	**3**
2	Gonopod femoral process present	***T.moniliformis***
–	Gonopod femoral process absent	***T.champasak* sp. nov.**
3	Gonopod femoral process absent	**4**
–	Gonopod femoral process present	**7**
4	Metaterga beset with long setae placed inside minute pores/knobs. Solenophore with vestigial parabasal lobe, distinct, acuminate, apical uncus and a couple of characteristic subapical outgrowths	***T.trichosa***
–	Metaterga with transverse rows of setae, instead of long hairs. Solenophore with or without a shoulder near base, and in a different shape	**5**
5	Metaterga with six rows of setae borne on small bosses	***T.hirsuta***
–	Metaterga with three rows of setae	**6**
6	Gonofemorite short. Solenophore without a basal shoulder	***T.cattiensis***
–	Gonofemorite considerably elongated. Solonophore with a basal shoulder	***T.peculiaris***
7	Gonofemorite carrying three processes	***T.pilosa***
–	Gonofemorite carrying only a single process	**8**
8	Femoral process long. Basal shoulder of solenophore well developed	***T.himalayaensis***
–	Femoral process short. Basal shoulder of solenophore less developed	***T.gracilis***

## ﻿Discussion

Attems (1937) distinguished the monotypic genus *Touranella* by a greatly shortened gonofemorite, the presence of a femoral process, and the densely setose metaterga. This diagnosis was supported by the discovery of the second species, *Touranellahimalayaensis* Golovatch, 1994. However, other *Touranella* species recently found in Vietnam have revealed new diagnostic characters as in Golovatch (2009a, 2009b, 2016) or Golovatch and Semenyuk (2010, 2018). Briefly, the genus can be recognized by having a submoniliform body, poorly developed paraterga, legs with neither modifications nor adenostyles, the presence of a sternal process between coxae 4, the gonofemorite either strongly reduced or very short as compared to the solenophore, the solenomere mostly rod-shaped or subflagelliform, sheathed by the solenophore distally, and both lamina medialis and lamina lateralis well developed.

Morphologically, the genus *Touranella* can be divided into two groups based on the presence or absence of the gonofemoral process. The first group includes the types species, *T.gracilis*, and four others, *T.himalayaensis*, *T.pilosa*, *T.trichosa*, and *T.moniliformis*. These species are characterized by the absence of the gonofemorite, or having it strongly reduced with a femoral process. They are also characterized by a solenophore with or without a lateral basal shoulder. The second group contains *T.peculiaris*, *T.cattiensis*, *T.hirsuta*, and *T.champasak* sp. nov., which are characterized by a very short or considerably elongated gonofemorite, without a femoral process. Given the absence of the femoral process and/or short gonofemorite, this second group is relatively close to the genus *Yuennanina* Attems, 1936. However, *Touranella* can be differentiated from *Yuennanina* using the first leg pair in males (femoral tubercles are absent from *Touranella* males, but present in *Yuennanina* males) and coxa (a thumb-like process is evident anteriorly in *Yuennanina*, but absent from *Touranella*). The relationship between *Touranella* with *Yuennanina* remains uncertain at this time.

The genus *Touranella* belongs to the tribe Alogolykini, created by Hoffman (1963: 591) for the genera *Tetracentrosternus* Pocock, 1895, *Alogolykus* Attems, 1936, and *Touranella*. He stated that members of this tribe could be recognized by having an extremely shortened gonofemorite; presence of a femoral process arising from the prefemorite; a slender solenophore that completely or partially sheaths the solenomere; and the first male leg pair without femoral tubercles. Jeekel (1965) noted the presence of femoral tubercles in the legs of *Tetracentrosternus* males and suggested a closer relationship between *Tetracentrosternus* and *Yuennanina*. Subsequently, Jeekel (1968: 127) retained these three genera (*Tetracentrosternus*, *Alogolykus*, and *Yuennanina*) in the tribe Alogolykini. Instead, he considered *Touranella* as *incertae sedis*, and stated that: “It is true that, as in all other Alogolykini, the gonopod femorite is reduced as in *Touranella*, but this is not a reason to postulate a close relationship. As a matter of fact, the tibiotarsus and its relation to the solenomerite rather strongly suggest the conditions in, e.g. the Orthomorphini, etc.” (Jeekel 1968: 65). This exclusion was still retained by Hoffman (1980: 171) and Jeekel (1980: 174). However, the genus was re-assigned to the tribe Alogolykini by Golovatch (1994: 187) and Nguyen and Sierwald (2013: 1179). This assignment was supported by additional newly described species (Golovatch 2009a, 2009b; Golovatch and Semenyuk 2010, 2018; Golovatch 2016).

According to Likhitrakarn et al. (2013) and Golovatch et al. (2021), the tribe Alogolykini can be distinguished from its close relative, Polydrepanini, as members have a strong, rod-shaped solenomere (vs a thin, flagelliform solenomere in Polydrepanini). Both these tribes are the only components of the subfamily Alogolykinae. This tribe Alogolykini currently consists of seven genera: a monotypic *Alogolykus* (from Myanmar), *Yuennanina* (three species from southern China), *Tetracentrosternus* (four species from Myanmar, Thailand, and southern China), and *Touranella* (eight species from Nepal, Laos, and Vietnam), *Singhalorthomorpha* Attems, 1914 (three species from Sri Lanka), a monotypic *Curiosoma* Golovatch, 1984 (from India), and finally a monotypic *Carlogonopus* Golovatch, Aswathy, Bhagirathan & Sudhikumar, 2021 (also from India) (Golovatch et al. 2021). However, a revision of this tribe is beyond the scope of this paper, and it is suggested that phylogenetic analyses employing morphological and molecular data are needed to elucidate relationships among these genera.

## Supplementary Material

XML Treatment for Touranella

XML Treatment for Touranella champasak

## References

[B1] Attems C (1914) Die indo-australischen Myriopoden. Archiv für Naturgeschichte 80A: 1–398.

[B2] Attems C (1936) Diplopoda of India. Memoirs of the Indian Museum 11(4): 133–323.

[B3] Attems C (1937) Myriapoda 3. Polydesmoidea I. Fam. Strongylosomidae. Das Tierreich 68: 1–300. 10.1515/9783111567099

[B4] Attems C (1938) Die von Dr. C. Dawydoff in französisch Indochina gesammelten Myriopoden. Mémoires du Muséum national d’histoire naturelle N.S. 6(2): 187–353.

[B5] Golovatch SI (1984) Some new or less known Paradoxosomatidae (Diplopoda: Polydesmida) from India. Acta Zoologica Hungarica 30(3–4): 327–353. 10.15298/arthsel.30.4.04

[B6] Golovatch SI (1994) Diplopoda from the Himalayas. Two new Alogolykini (Polydesmida: Paradoxosomatidae). Senckenbergiana Biologica 73(1–2): 183–187.

[B7] Golovatch SI (2009a) On several new or poorly-known Oriental Paradoxosomatidae (Diplopoda: Polydesmida), IX. Arthropoda Selecta 18(3/4): 119–124.

[B8] Golovatch SI (2009b) On several new or poorly-known Oriental Paradoxosomatidae (Diplopoda: Polydesmida), VIII. Arthropoda Selecta 18(1/2): 1–7. 10.15298/arthsel.19.3.02

[B9] Golovatch SI (2016) On several new or poorly-known Oriental Paradoxosomatidae (Diplopoda, Polydesmida), XIX. Arthropoda Selecta 25(2): 131–152. 10.15298/arthsel.25.2.01

[B10] Golovatch SI Semenyuk II (2010) On several new or poorly-known Oriental Paradoxosomatidae (Diplopoda: Polydesmida), X. Arthropoda Selecta 19(3): 123–127. 10.15298/arthsel.19.3.02

[B11] Golovatch SI Semenyuk II (2018) On several new or poorly-known Oriental Paradoxosomatidae (Diplopoda: Polydesmida), XXIII. Arthropoda Selecta 27(1): 1–21. 10.15298/arthsel.31.1.01

[B12] Golovatch SI Aswathy MD Bhagirathan U Sudhikumar AV (2021) Review of the millipede tribe Polydrepanini, with the description of a new species from Kerala state, southern India (Diplopoda, Polydesmida, Paradoxosomatidae, Alogolykinae). Zootaxa 5068(4): 485–516. 10.11646/zootaxa.5068.4.2 34810694

[B13] Hoffman RL (1963) A contribution to the knowledge of Asiatic strongylosomoid Diplopoda (Polydesmida: Strongylosomatidae). Annals and Magazine of Natural History (Series 13) 5: 577–593. 10.1080/00222936208651289

[B14] Hoffman RL (1980) Classification of the Diplopoda. Genève, 1–237.

[B15] Jeekel CAW (1965) A revision of the Burmese Paradoxosomatidae (Diplopoda, Polydesmida) in the Museo Civico di Storia Naturale at Genoa (Part I). Tijdschrift voor Entomologie 108: 95–144.

[B16] Jeekel CAW (1968) On the classification and geographical distribution of the family Paradoxosomatidae (Diplopoda, Polydesmida). Thesis, University of Amsterdam, Rotterdam, 162 pp.

[B17] Jeekel CAW (1980) On some little known Paradoxosomatidae from India and Ceylon, with the description of four new genera (Diplopoda, Polydesmida). Beaufortia 30(8): 163–178.

[B18] Likhitrakarn N Golovatch SI Panha S (2013) The millipede genus *Tetracentrosternus* Pocock, 1895 (Polydesmida, Paradoxosomatidae, Alogolykinae, Alogolykini), with a description of the first, new species from Thailand. ZooKeys 358: 1–10. 10.3897/zookeys.358.6582 PMC3867176 24363581

[B19] Nguyen AD Sierwald P (2013) A worldwide catalog of the family Paradoxosomatidae Daday, 1889 (Diplopoda: Polydesmida). Check List 9(6): 1132–1353. 10.15560/9.6.1132

[B20] Sierwald P Spelda J (2021) MilliBase. *Touranella* Attems, 1937. http://www.millibase.org/aphia.php?p=taxdetails&id=892789 [Accessed on 2022-11-08]

